# Impact of preserving circumflex iliac nodes distal to the external iliac nodes (CINDEIN) on prognosis in low-risk endometrial cancer patients

**DOI:** 10.1016/j.gore.2026.102181

**Published:** 2026-08-05

**Authors:** Yasushi Yamada, Hisanori Kobara, Masako Nakajima, Hirofumi Ando, Tsutomu Miyamoto

**Affiliations:** a Department of Obstetrics and Gynecology, Shinshu University School of Medicine, Matsumoto, Japan; b Department of Komagane Kogen Ladies Clinic, 759-195 Akaho, Komagane, Japan

**Keywords:** CINDEIN, Endometrial cancer, Pelvic lymphadenectomy, Lymphedema, Prognosis

## Abstract

**Aim:**

Sentinel lymph node (SLN) mapping has become the standard approach for lymph node (LN) assessment in patients with low-risk endometrial cancer (EC). However, until its coverage by the Japanese national health insurance system in 2025, pelvic lymphadenectomy (PLA) had been routinely performed for nodal assessment in Japan. The resection of circumflex iliac nodes distal to the external iliac nodes (CINDEIN) in PLA is associated with an increased risk of lymphedema. CINDEIN preservation in EC patients reduces the risk of lymphedema; however, the impact of CINDEIN preservation on recurrence patterns and prognosis is unclear. Therefore, we herein examined the effects of CINDEIN preservation on recurrence patterns, survival outcomes, and lymphedema in patients with low-risk EC.

**Methods:**

We retrospectively analyzed 65 patients with preoperatively diagnosed low-risk EC who underwent PLA as their initial treatment between January 2016 and December 2024. Patients were divided into two groups according to whether CINDEIN was resected or preserved, and treatment effects and prognosis were evaluated.

Results: Lymphedema was noted in 1 case in the CINDEIN-preserved group (2.7%) and in 7 in the CINDEIN-resected group (25%), demonstrating a significant difference (*P* = 0.017). Recurrence was noted in 4 cases in the CINDEIN-preserved group and in 2 in the CINDEIN-resected group (*P* > 0.05). There were no significant differences in PFS or OS between the two groups.

**Conclusions:**

CINDEIN preservation may be performed on patients with low-risk EC without worsening their prognosis and correlated with a reduced risk of lymphedema.

## Highlights

• CINDEIN preservation during PLA is associated with reduced incidence of lymphedema.
• CINDEIN preservation does not compromise oncological outcomes in low-risk EC.
• CINDEIN preservation may represent a viable treatment strategy during PLA.

## 1 Introduction

In Japan, the incidence of endometrial cancer (EC) is the highest among gynecological cancers and has been increasing yearly; the number of cases is estimated to have increased 13-fold over the past 40 years, according to the database of the National Cancer Center Institute for Cancer Control (National Cancer Center, 2026). The primary treatment for EC is surgery. The standard procedures include total hysterectomy (TH), bilateral salpingo-oophorectomy (BSO), and retroperitoneal lymph node (LN) assessment; for the latter, sentinel lymph node (SLN) mapping is recommended over lymphadenectomy (NCCN, 2026). Since the presence of LN metastasis is a high-risk factor for recurrence, it is important to reliably identify metastasis-positive cases through LN dissection and initiate postoperative adjuvant therapy to improve prognosis (Tang et al., 1998; Versluis et al., 2018). Because SLN mapping was not covered by the Japanese national health insurance system until 2025, pelvic lymphadenectomy (PLA) was the standard procedure for the LN assessment in patients with low-risk EC, defined as having less than 50% myometrial invasion and a histological type of endometrioid carcinoma, grade 1 or 2 (NCCN, 2026). Furthermore, because para-aortic lymph node assessment, including para-aortic lymphadenectomy (PAoLA), is recommended only for patients with high-risk EC, PAoLA is not routinely performed in patients with low-risk EC at our institution (NCCN, 2026). The complications of PLA, such as lymphedema (28%), lymphocyst (9%), and thrombosis (2%), pose significant challenges (Konno et al., 2021). Among them, lymphedema following surgery for gynecological cancer often involves pain and discomfort and may be permanent; therefore, it reduces quality of life and negatively affects the body image of gynecological cancer patients (Seki and Okutsu, 2023).

Conventionally, PLA was performed for LN assessment in patients with low-risk EC to obtain accurate prognostic information (NCCN, 2026). However, the Japan Society of Gynecologic Oncology (JSGO) Guidelines recommend omitting not only PAoLA but also PLA in patients with low-risk EC, based on evidence from a phase III randomized trial demonstrating that PLA does not improve the prognosis of patients with early-stage EC (JSGO, 2023; Benedetti Panici et al., 2008). The incidence of pelvic lymph node (PLN) metastasis was previously reported to be 1.6% in patients with stage I low-grade EC (Nayyar et al., 2018). However, it was also shown to be higher in low-risk EC patients with less than 50% myometrial invasion and/or a tumor diameter ≥ 2 cm than in those without myometrial invasion and with a tumor diameter < 2 cm (Mariani et al., 2000). Therefore, although PLA is currently optional, it has been recommended at our hospital for low-risk EC patients with less than 50% myometrial invasion and a tumor diameter ≥ 2 cm.

Circumflex iliac nodes distal to the external iliac nodes (CINDEIN) are among the regional LNs of EC and are generally removed during PLA. However, there are various opinions regarding the necessity of their removal because the resection of CINDEIN was found to contribute to the risk of lymphedema (Todo et al., 2010). Based on these opinions and in an attempt to reduce the risk of lymphedema when performing PLA on low-risk EC patients, we have preserved CINDEIN since 2020. Although CINDEIN preservation has been shown to effectively decrease lymphedema, its impact on the prognosis of patients remains unknown. Therefore, we herein investigated the impact of CINDEIN preservation on recurrence patterns, survival outcomes, and lymphedema in patients with low-risk EC.

## 2 Patients and methods

### 2.1 Patients

**Fig. 1:**
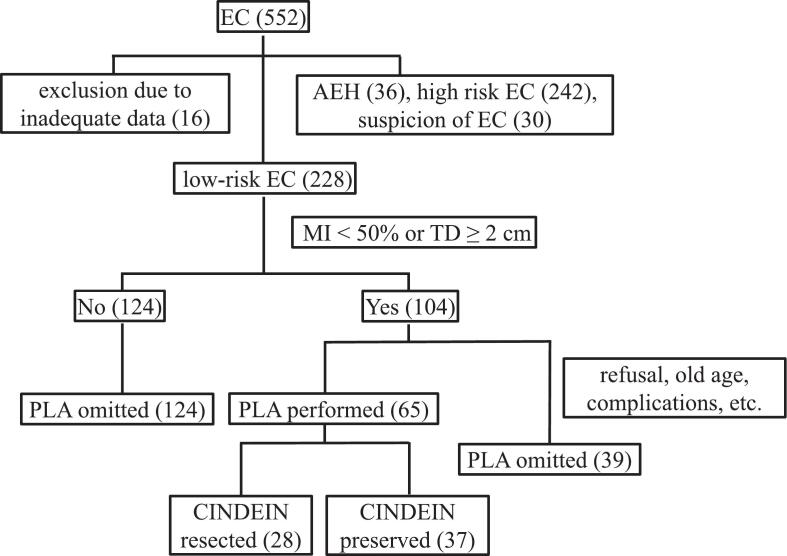
Profile of this study.

A total of 552 patients with EC who underwent primary surgical treatment at Shinshu University Hospital between January 2016 and December 2024 were initially reviewed. Of these, 324 patients were excluded because of a preoperative diagnosis of atypical endometrial hyperplasia, high-risk EC, or suspected EC based solely on imaging, or because of inadequate data. At our institution, patients with ≥50% myometrial invasion or high-risk histological subtypes (such as serous or clear cell carcinoma) were classified as having high-risk EC and underwent PAoLA in addition to PLA. The remaining 228 patients were preoperatively classified as having low-risk EC, and PAoLA was omitted in all of them. In our department, PLA was indicated for low-risk EC patients with <50% myometrial invasion and a tumor diameter ≥ 2 cm. Among the low-risk patients, 124 had a tumor diameter < 2 cm and no myometrial invasion. The remaining 104 patients had <50% myometrial invasion and/or a tumor diameter ≥ 2 cm; of these, 65 underwent PLA and 39 did not undergo PLA due to patient refusal, advanced age, or severe complications (Fig. 1). Clinical and pathological characteristics, surgical outcomes, and prognosis were retrospectively reviewed from medical records.

### 2.2 Surgical technique

Regional LNs for EC include PLNs, which are the obturator nodes (ON), circumflex iliac nodes distal to the obturator nodes (CINDON), external iliac nodes (EIN), CINDEIN, internal iliac nodes (IIN), common iliac nodes (CIN), sacral nodes (SN), parametrial nodes (PN), and para-aortic LNs (JSGO, 2022) (Supplementary fig. 1). Systemic PLA, which includes ON, EIN, CINDEIN, IIN, CINDON, and CIN among the aforementioned regional LNs, was performed after TH and BSO. CINDEIN were resected in all patients from 2016 to 2019. In contrast, CINDEIN were routinely preserved between 2020 and 2024, except in patients whose CINDEIN showed enlargement on preoperative magnetic resonance imaging or during intraoperative inspection/palpation. In these patients, CINDEIN were resected on the enlarged side and preserved on the opposite side. SN and PN were not resected in all cases. The stumps of the lymphatic vessels of CINDON were clipped or ligated and then divided using a vessel sealing device, such as LigaSure™ (Medtronic, Dublin, Ireland). In contrast, lymphatic vessels other than those of CINDON were divided using the vessel sealing device alone, without mandatory clipping or ligation. PLNs were not removed via *en bloc* resection but rather by the resection of individual LN stations. After PLA, the retroperitoneum was not closed and an antiadhesive material was applied. A drain was placed in the pouch of Douglas, which was removed after a few days if there was no postoperative bleeding. The operative time was calculated from the initial skin incision to the completion of all surgical procedures.

### 2.3 Postoperative adjuvant therapy and follow-up

If risk factors for recurrence, such as lymphovascular space invasion (LVSI), deep myometrial invasion, LN metastasis, adnexal metastasis, or high-grade histological subtypes, were identified on pathological examination, patients received 3–6 cycles of adjuvant chemotherapy. Neither adjuvant radiotherapy nor additional PAoLA was performed. Patients were followed up every 3 months for the first 3 years, every 6 months from 4 to 6 years, and annually thereafter.

### 2.4 Assessment of lymphedema

During adjuvant therapy and routine postoperative follow-up visits, lymphedema was assessed via medical interviews, inspection, and palpation. If pitting edema and asymmetry between the left and right lower extremities, inguinal regions, and lower abdomen were observed, lymphedema was diagnosed. Lymphedema was clinically staged based on the 2020 International Society of Lymphology clinical staging criteria (Executive Committee of the International Society of Lymphology, 2020). Stage 0 (latent or subclinical) represents a condition with impaired lymph transport but without visible swelling. Stage 1 is characterized by an early accumulation of protein-rich fluid that decreases upon limb elevation, potentially accompanied by pitting. In Stage 2, limb elevation alone rarely diminishes tissue enlargement, and pitting is consistently present. Stage 3, or lymphostatic elephantiasis, is defined by the potential absence of pitting alongside severe trophic skin changes, such as acanthosis, altered skin thickness, fat and fibrosis deposition, and warty overgrowths.

### 2.5 Outcome

The primary outcome measure was the incidence of postoperative lymphedema. The secondary outcome measures were overall survival (OS), progression-free survival (PFS), and the recurrence rate.

### 2.6 Statistical analyses

All statistical analyses were performed using EZR version 1.68 (Jichi Medical University, Tochigi, Japan), which is a graphical user interface for R (The R Foundation for Statistical Computing, Vienna, Austria). More precisely, it is a modified version of R commander designed to add statistical functions frequently used in biostatistics (Kanda, 2013). The Mann-Whitney *U* test and Fisher's exact test were used to compare continuous and categorical variables, respectively. Survival curves for overall survival (OS) and progression-free survival (PFS) were plotted using the Kaplan-Meier method, and differences were evaluated using the Log-rank test. A multivariable analysis was performed to evaluate risk factors for lymphedema following PLA. The significance of differences was defined as *P* < 0.05.

### 2.7 Ethical review

The present study was approved by the Ethical Committee for Medical and Biological Research of Shinshu University School of Medicine (approval number 6540). Due to the retrospective design of the study, the requirement for written informed consent was waived, and an opt-out consent process was implemented. Information about the study was made available on the institutional website, and participants were given the opportunity to opt out of the study.

## 3 Results

### 3.1 Patient characteristics

Sixty-five patients were performed PLA; 37 who underwent CINDEIN preservation were classified as the CINDEIN-preserved group and 28 who underwent CINDEIN resection as the CINDEIN-resected group. Table 1 summarizes patient characteristics. No significant differences were observed between the two groups in terms of age, body mass index (BMI), preoperative cancer antigen (CA) 125 levels, which is useful for early detection of recurrence in EC and recommended for surveillance if initially elevated (NCCN, 2026), the FIGO stage, pathological characteristics, the number of recurrences, or disease-specific deaths between the two groups (*P* > 0.05). LN metastasis was noted in four patients (10.8%) in the CINDEIN-preserved group and in three (10.7%) in the CINDEIN-resected group; however, no histological metastasis to CINDEIN itself was observed in the CINDEIN-resected group. Cases that upstaged from preoperatively low-risk to postoperatively high-risk included 14 (37.8%) patients in the CINDEIN-preserved group and 10 (35.7%) patients in the CINDEIN-resected group (*P* = 1.000). Since CINDEIN preservation has been performed since 2020, the median follow-up period of the CINDEIN-resected group was significantly longer than that of the CINDEIN-preserved group (57.0 and 41.0 months, respectively, *P* = 0.006). Although recurrence was detected in four patients in the CINDEIN-preserved group (10.8%), there were no cases of recurrence involving CINDEIN. Recurrence was observed in two patients (7.1%) in the CINDEIN-resected group (*P* > 0.05).

**Table 1:** Clinical and pathological characteristics of CINDEIN-preserved and CINDEIN-resected groups.

	CINDEIN-preserved group (*n* = 37)	CINDEIN-resected group (*n* = 28)	*P* value
			
Age, median (range), years	62 (41–79)	57 (48–76)	0.569
BMI, median (range), kg/m^2^	23.8 (19.2–32.9)	23.7 (18.0–34.6)	0.942
Preoperative CA125, median (range), U/ml	21.0 (7.7–389.6)	22.5 (12.6–442.7)	0.314
FIGO stage (2008), n (%)			
IA	23 (62.2)	21 (75.0)	0.299
IB	10 (27.0)	3 (10.7)	
II	0 (0)	1 (3.6)	
IIIA	0 (0)	0 (0)	
IIIB	0 (0)	0 (0)	
IIIC1	4 (10.8)	3 (10.7)	
High-grade histological types, n (%)	1 (2.7)	3 (10.7)	0.307
Deep myometrial invasion, n (%)	11 (29.7)	4 (14.3)	0.234
Cervical involvement, n (%)	1 (2.7)	1 (3.6)	1.000
LVSI, n (%)	12 (32.4)	9 (32.1)	1.000
PLN metastasis, n (%)	4 (10.8)	3 (10.7)	1.000
	ON 4, EIN 1, IIN 1	CIN 1, EIN 3	
Adnexal metastasis, n (%)	0 (0)	0 (0)	
Positive PC, n (%)	1 (2.7)	2 (7.1)	0.573
Adjuvant chemotherapy, n (%)	16 (43.2)	10 (35.7)	0.614
Recurrence, n (%)	4 (10.8)	2 (7.1)	0.692
Site of recurrence, n (%)	lung 3 (8.1)	PAN 1 (3.6)	
	PAN 1 (2.7)	vaginal 1 (3.6)	
	metastatic 1 (2.7)		
Disease-specific death, n (%)	1 (2.7)	0 (0)	1.000
Observation period, median (range), months	41.0 (7–81)	57.0 (8–107)	0.006^⁎⁎^

CINDEIN: circumflex iliac nodes distal to the external iliac nodes, BMI: body mass index, CA125: carcinoma antigen 125, LVSI: lymph-vascular space invasion, PLN: pelvic lymph node, ON: obturator nodes, EIN: external iliac nodes, IIN: internal iliac nodes, CIN: common iliac nodes, PC: peritoneal washing cytology, PAN: para-aortic lymph node ** *p* < 0.01.

### 3.2 Surgical outcomes and complications

Table 2 shows the surgical outcomes of the two groups, and Supplementary table 1 shows the characteristics of lymphedema cases. The rate of MIS in the CINDEIN-preserved group was significantly higher than that in the CINDEIN-resected group (86.5% vs. 39.3%, *P* < 0.001). Reflecting this, the CINDEIN-preserved group associated with a longer operating time, a longer postoperative hospital stay, and less blood loss (*P* = 0.048, *P* = 0.014, and P < 0.001, respectively). Lymphedema was observed in 1 patient in the CINDEIN-preserved group (2.7%) and in seven in the CINDEIN-resected group (25.0%), demonstrating a significant difference (*P* = 0.017) (Table 2). The median onset time of lymphedema was 3.5 months (range, 1–97 months). In the CINDEIN-resected group, three patients developed severe lymphedema that was refractory to conservative treatment, two of whom underwent lymphatic-venous anastomosis (LVA). In addition, lymphedema occurred exclusively on the side where CINDEIN had been resected in all affected patients in the CINDEIN-resected group. In contrast, there was only one patient with lymphedema in the CINDEIN-preserved group, which spontaneously resolved by only conservative treatment (Supplementary Table 1). There were significant differences in the rates of minimal invasive surgery (MIS), blood loss, operating time, and postoperative hospital stay between the two groups, but not in the number of resected LNs or postoperative complications, except for lymphedema (Table 2).

**Table 2:** Surgical outcomes of CINDEIN-preserved and CINDEIN-resected groups.

	CINDEIN-preserved group (n = 37)	CINDEIN-resected group (n = 28)	P value
Surgical procedure, n (%)			
Laparotomy	5 (13.5)	17 (60.7)	<0.001^⁎⁎⁎^
MIS	32 (86.5)	11 (39.3)	
Operating time, median (range), minutes	356 (251–668)	334 (215–431)	0.048^⁎^
Blood loss, median (range), ml	50 (10–280)	135 (10–750)	0.014^⁎^
Number of resected LNs, median (range)	29 (13–57)	34.5 (19–60)	0.099
Number of resected CINDEIN, median (range)	N/A	4 (0−11)	
Postoperative complications, n (%)			
Lymphedema	1 (2.7)	7 (25)	0.017^⁎^
Thrombosis	3 (8.1)	4 (14.3)	0.453
Lower extremity nerve injury	2 (5.4)	1 (3.6)	1.000
Infection	0 (0)	3 (10.7)	0.075
Bleeding	1 (2.7)	0 (0)	1.000
Ileus	3 (8.1)	1 (3.6)	0.628
Postoperative hospital stay, median (range), days	5 (3−13)	7 (3−12)	<0.001^⁎⁎⁎^

CINDEIN: circumflex iliac nodes distal to the external iliac nodes, MIS: minimal invasive surgery, LN: lymph node, N/A: not applicable * p < 0.05 *** *p* < 0.001.

Survival and risk factors for lymphedema.

**Fig. 2:**
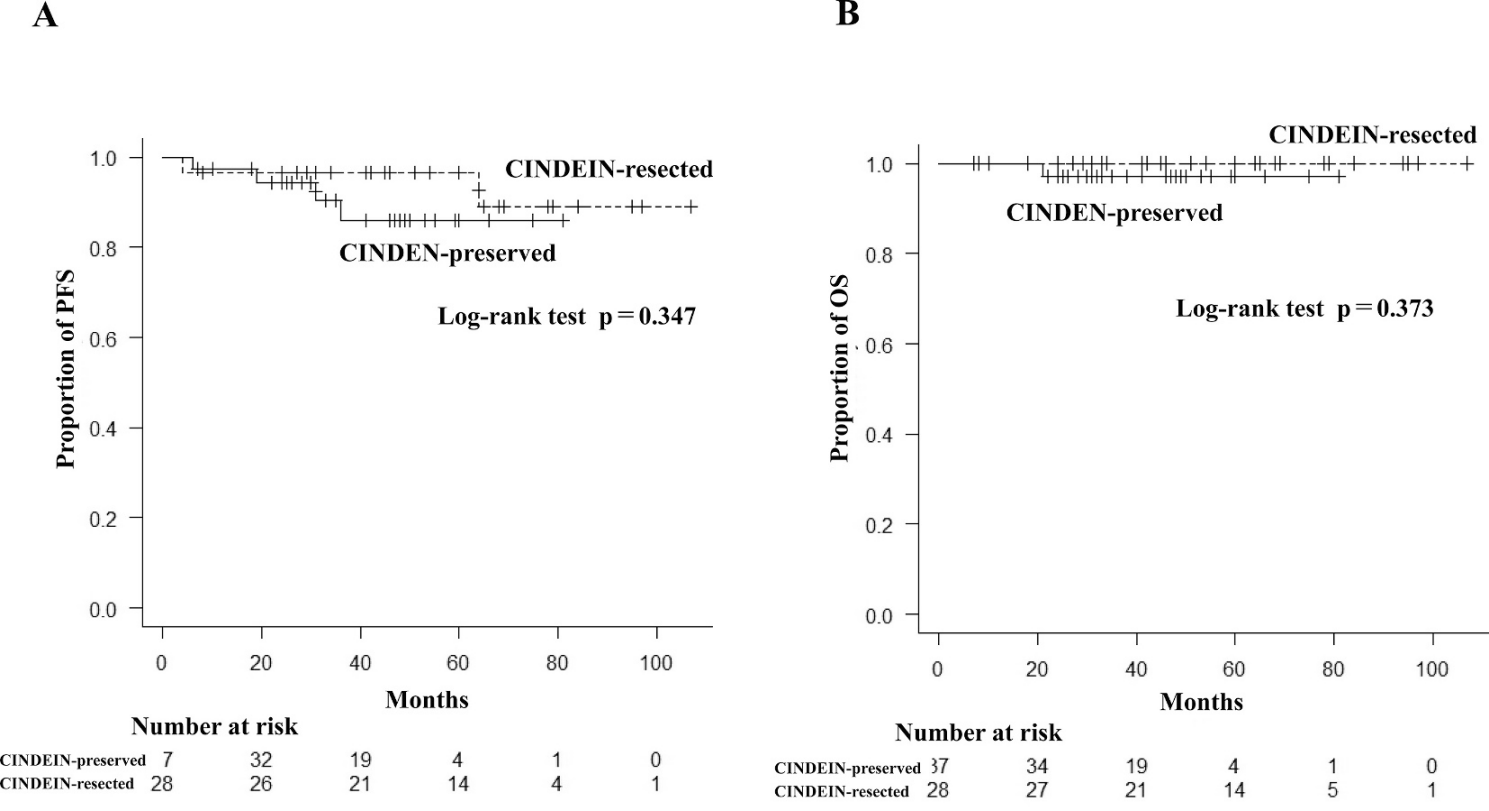
Progression-free survival (PFS) and overall survival (OS).

Regarding oncological outcomes, there were no significant differences in OS or PFS between the two groups (Fig. 2). A multivariable logistic regression analysis confirmed that CINDEIN resection was an independent risk factor for the development of lymphedema (odds ratio 15.8, 95% confidence interval 1.540–161.0, *P* = 0.02) (Table 3).

**Table 3:** Multivariate analysis of risk factors for lymphedema.

factor	odds ratio (95% CI)	P value
Number of resected LNs		
<32	1	
≥32	0.446 (0.075–2.640)	0.374
Surgical procedure		
MIS	1	
laparotomy	1.020 (0.166–6.110)	0.995
Resected CINDEIN		
No	1	
Yes	15.800 (1.540–161.0)	0.02^⁎^

A multivariate analysis identified CINDEIN resection as an independent risk factor for lymphedema.

CI: confidence interval, LN: lymph node, MIS: minimal invasive surgery, CINDEIN: circumflex iliac nodes distal to the external iliac nodes * p < 0.05.

## 4 Discussion

In the present study, the incidence of lymphedema was significantly decreased by the preservation of CINDEIN, and no significant differences were observed in OS or PFS between the CINDEN-preserved and CINDEIN-resected groups. To the best of our knowledge, this is the first study to examine recurrence, OS, and PFS following CINDEIN preservation in patients with low-risk EC.

EC frequently develops after menopause. Since abnormal vaginal bleeding is typically recognized from its onset, EC is often diagnosed at an early stage, and the rate of low-risk EC is approximately 60% (Zola et al., 2022). The LN assessment in low-risk EC patients is important for obtaining accurate prognostic information, and PLA is one of the standard methods conducted for this purpose (Tang et al., 1998, Versluis et al., 2018). Clinical trials reported the utility of PLA for surgical staging; however, it did not improve the prognosis of low-risk EC patients (Benedetti Panici et al., 2008, Kitchener et al., 2009). Therefore, the omission of PLA is now suggested for low-risk EC patients according to the JSGO guidelines, as mentioned above (JSGO, 2023). SLN mapping, which decreases the incidence of lymphedema more than PLA (Ballester et al., 2011), is increasingly being recommended based on the National Comprehensive Cancer Network (NCCN) guidelines (NCCN, 2026). In Japan, SLN mapping for EC had been limited to select institutions as a non-insured treatment until 2025. However, it was covered by the national health insurance system in June 2026, and we introduced this procedure in our department in July 2026. SLN mapping is expected to be widely adopted across many Japanese gynecologic oncology departments in the near future, consistent with global trends. Nevertheless, SLN detection fails in approximately 10% to 20% of cases (Ballester et al., 2011; Togami et al., 2022). In such cases of failed mapping, the NCCN guidelines recommend performing an ipsilateral pelvic lymph node dissection (NCCN, 2026). In patients with failed SLN mapping, or in institutions where SLN mapping remains unavailable, preservation of the CINDEIN may contribute to reducing lymphedema, as suggested by our present and previous findings (Todo et al., 2010; Wang et al., 2024).

Although PLA is widely performed, the extent of regional LN dissection and the specific procedures performed vary among institutions, as demonstrated in the literature. In an Italian study, resected regional LNs for EC comprised EIN, ON, IIN, and CIN (Benedetti Panici et al., 2008). Konno et al. added CINDEIN and CINDON (Konno et al., 2011), while Wang et al. included CINDEIN, CINDON, and PN (Wang et al., 2024). Although CINDEIN are classified as regional LNs for EC, the rate of metastasis is extremely low. Odagiri et al. showed that CINDEIN metastasis was not detected in 266 patients with EC who underwent PLA and PAoLA (Odagiri et al., 2014). Furthermore, Togami et al. found that during SLN mapping in 122 patients with preoperative FIGO stage IA EC, CINDEIN was not identified as a SLN in any patient (Togami et al., 2022). In the FIRES Trial, CINDEIN was not detected as SLN in stage I EC patients (Rossi et al., 2017). Based on these findings, the preservation of CINDEIN needs to be considered.

CINDEIN resection contributes to lymphedema because CINDEIN are an enlarged lobular conglomerate of lymphatic channel-rich nodal and adipose tissue. Within this tissue, lymphatic vessels pass into the iliolumbar muscle, acting as a direct extension of the more distally located deep inguinal nodes or Cloquet's node in the groin, which drain the lower extremities (Abu-Rustum and Barakat, 2007). We selected “resected CINDEIN”, “surgical procedure”, and “number of resected LNs” as risk factors for lymphedema. The reason for selecting “surgical procedure” was that the MIS rate was higher in the CINDEIN-preserved group. Todo et al. reported that a large number of resected LNs was risk factor for lymphedema (Todo et al., 2010). Consequently, multivariable logistic regression analysis indicated that CINDEIN resection was an independent risk factor for the development of lymphedema. Interestingly, lower extremity lymphedema was not observed in the legs where the CINDEIN was preserved among patients who underwent unilateral CINDEIN resection. Moreover, patients with persistent lymphedema or those requiring LVA were exclusively in the CINDEIN-resected group. These results suggest that the resection of CINDEIN correlates not only with the occurrence, but also with the severity of lymphedema.

The rate of MIS was higher in the CINDEIN-preserved group than in the CINDEIN-resected group, reflecting the adoption of CINDEIN preservation since 2020. In the CINDEIN-preserved group, the operative time was longer, while the volume of bleeding was lower and the postoperative hospital stay was shorter than those in the CINDEIN-resected group. These results may be associated with the difference observed in the MIS rate. The rate of lymphedema in or the prognosis of EC generally does not significantly differ based on the type of surgical procedure performed (open, laparoscopy, or robotic surgery) (Padmanabhan et al., 2019). Therefore, the difference observed in the MIS rate between the two groups in the present study likely had a negligible or no effect on the results obtained.

According to the present results and previous findings (Todo et al., 2010, Wang et al., 2024), the preservation of CINDEIN in low-risk EC patients may reduce the risk of lymphedema without worsening their prognosis. However, since limited information is currently available on the prognosis of CINDEIN preservation in high-risk EC patients, a careful decision is needed when performing this procedure on these patients.

This study has two major limitations. First, the sample size was small due to its single-center retrospective design. Furthermore, there may be selection and confounding biases. Therefore, the present results should be interpreted with caution.

In summary, CINDEIN preservation during PLA may be performed without worsening the prognosis of low-risk EC patients and may reduce the risk of lymphedema.

## Declaration of generative AI and AI-assisted technologies in the manuscript preparation process

Statement: During the preparation of this work the authors used ChatGPT in order to create Supplementary Fig. 1. After using this tool/service, the authors reviewed and edited the content as needed and take full responsibility for the content of the published article.

## CRediT authorship contribution statement

**Yasushi Yamada:** Writing – original draft. **Hisanori Kobara:** Writing – review & editing. **Masako Nakajima:** Investigation. **Hirofumi Ando:** Investigation. **Tsutomu Miyamoto:** Supervision.

## Declaration of competing interest

The authors declare that they have no known competing financial interests or personal relationships that could have appeared to influence the work reported in this paper.

## Appendix A Supplementary data

Supplementary material 1: Supplementary Figure 1 Characteristics of lymphedema cases

Supplementary material 2: Supplementary Table 1 Locations of the regional lymph nodes for endometrial cancer. A: para-aortic lymph nodes. B: common iliac nodes (CIN). C: external iliac nodes (EIN). D: circumflex iliac nodes distal to the external iliac nodes (CINDEIN). E: sacral nodes (SN). F: internal iliac nodes (IIN). G: obturator nodes (ON). H: circumflex iliac nodes distal to the obturator nodes (CINDON). I: parametrial nodes (PN).
